# Facial aesthetic evaluation in patients with repaired cleft lip and palate

**DOI:** 10.1186/s40902-019-0205-5

**Published:** 2019-06-04

**Authors:** Tatiana S. Paiva, Marcia Andre, Beatriz Silva Mattos

**Affiliations:** Department of Surgery, Prosthesis and Maxillofacial Traumatology, University of São Paulo Dentistry School, 306 Mato Grosso Street, Office 1301, São Paulo, 01239040 Brazil

**Keywords:** Assessment, Cleft lip palate, Facial appearance, Laypeople

We have read with great interest the recent paper by Alhayek et al. [1]. The aesthetic aspects of the nasolabial region are one of the most important instruments to evaluate the success of the treatment of patients with cleft lip and palate. Although there are many surgical protocols for cleft reconstruction, the results are usually analysed subjectively because they depend on the observer’s particular opinion, ethnic, cultural and age patterns, and it is therefore difficult to measure them [2].

It is important that professionals treating patients with cleft lip and palate are aware of aesthetic assessment methods for analysis of results. However, this analysis has different models and combinations of evaluators and may use ordinal or analogue visual scales [3], although subjective methods are the ones that best reflect the population’s perception regarding the patient’s facial impairment [4].

The study by Alhayek et al. [1] evaluates the aesthetic perception of the cleft lip and palate using different groups of evaluators. These evaluations are important for treatment planning, as well as assisting in the reevaluation of rehabilitation protocols for clefts of the lip and palate, considering the important psychosocial limitations, anxiety and depression experienced by these individuals [5–7].

Another important aspect of the study by Alhayek et al. [1] is the use of a large number of evaluators both in the group of health professionals and in the lay group. It is also worth noting the use in the group of professionals of three different specialties showing the different perceptions in their areas. However, we did not see if the evaluators did a previous calibration, such as the orientation of the topics to be evaluated (scar quality, asymmetries, lip volume, etc.). This is especially important because even though it is a subjective evaluation, this standardization contributes to the reliability of the results, as well as to the reproducibility of the study in other populations.

In addition to questions related to the aesthetics observed in the photographs, the evaluators answered two questions about the perception of the influence of cleft lip and palate on the social interactions and on the professional life. Questions related to quality of life have more closeness to the reality experienced when made to the patient himself. For what would be the criteria for the evaluator to answer about the influence of the cleft in the professional and social life of the five children?

Alhayek et al. [1] used four types of photographic positions (frontal, right lateral, right side and smile). Thus, it is not possible to understand why the right side was used exclusively. Is all unilateral cleft on the right side? And about bilateral cleft, did the right side always have the same standard in this sample?

The authors also report that discrepancies in the perception of facial aesthetics among evaluators could be attributed to different modalities and treatment protocols; however, it does not seem appropriate to conclude this using a small number of patients. These small caveats, however, do not take away the main relevant messages and discussion raised by Alhayek et al. [1].
